# Supplementary figures and images for: Supplier-origin gut microbiomes affect host body weight and select autism-related behaviors
Source: Gut Microbes. 2024 Aug 6;16(1):2385524. doi: 10.1080/19490976.2024.2385524 (PMC11305029; doi:10.1080/19490976.2024.2385524)

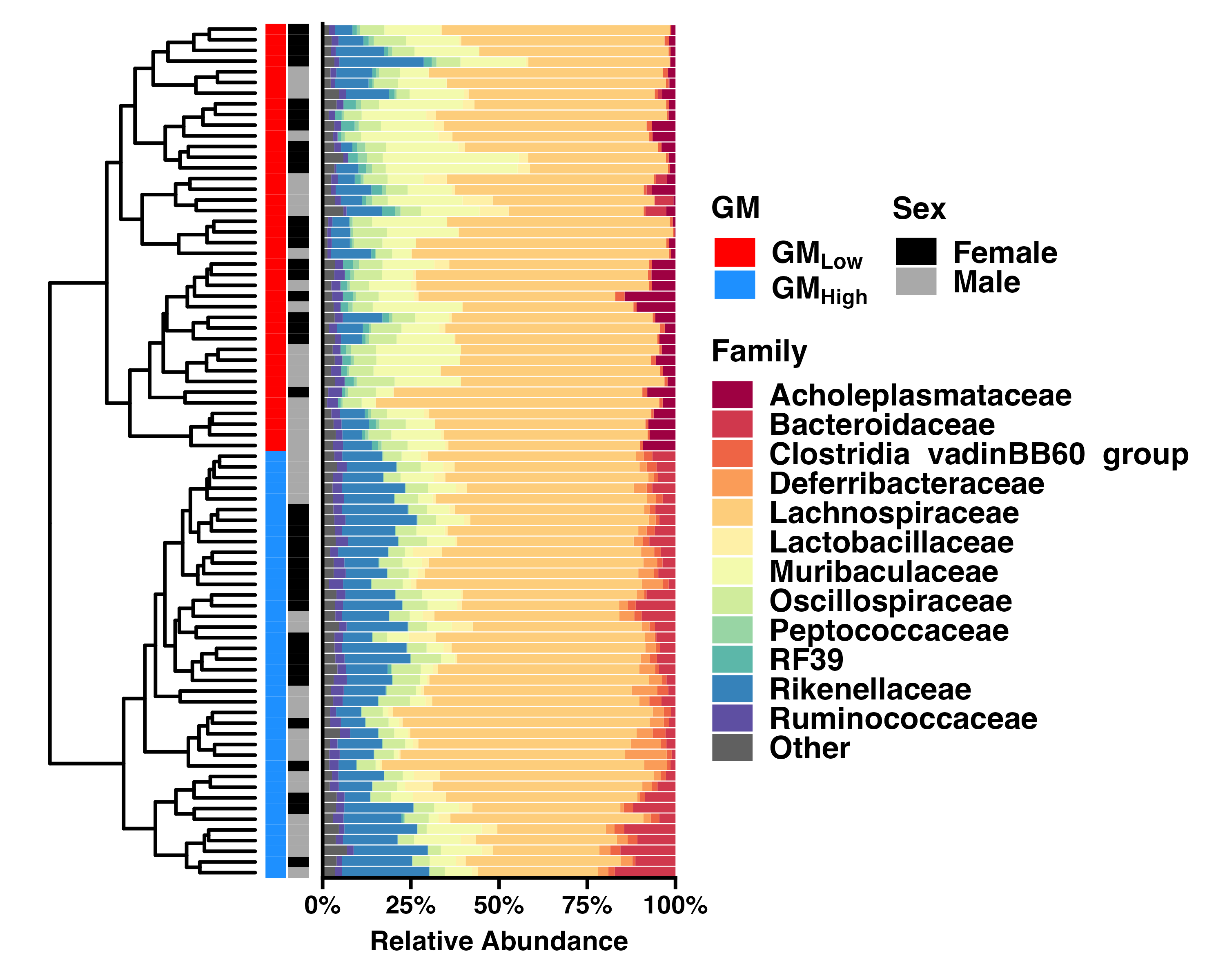

Supplement: Supplemental Material [file KGMI_A_2385524_SM3678.zip › Figure S1.tiff]

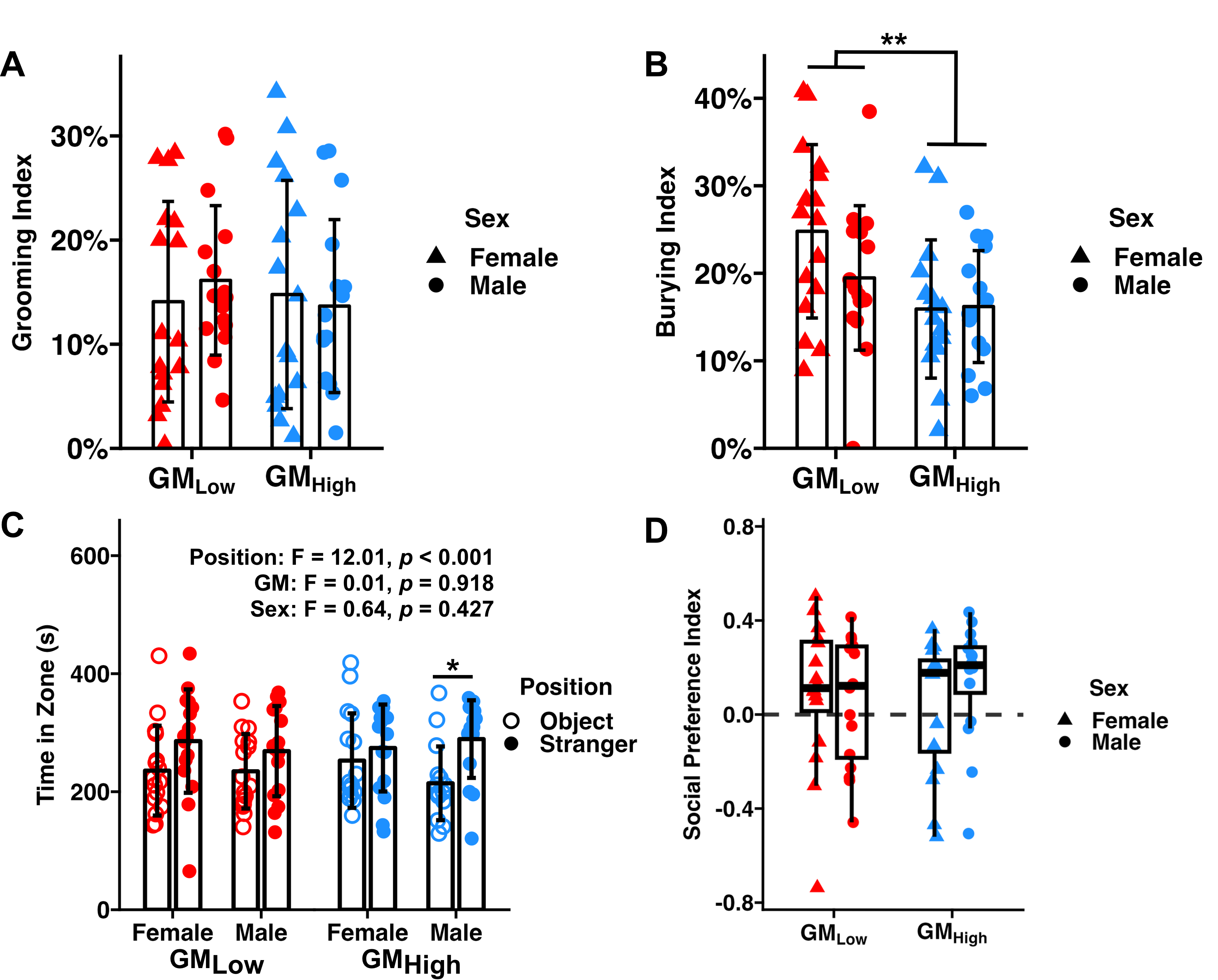

Supplement: Supplemental Material [file KGMI_A_2385524_SM3678.zip › Figure S2.tiff]

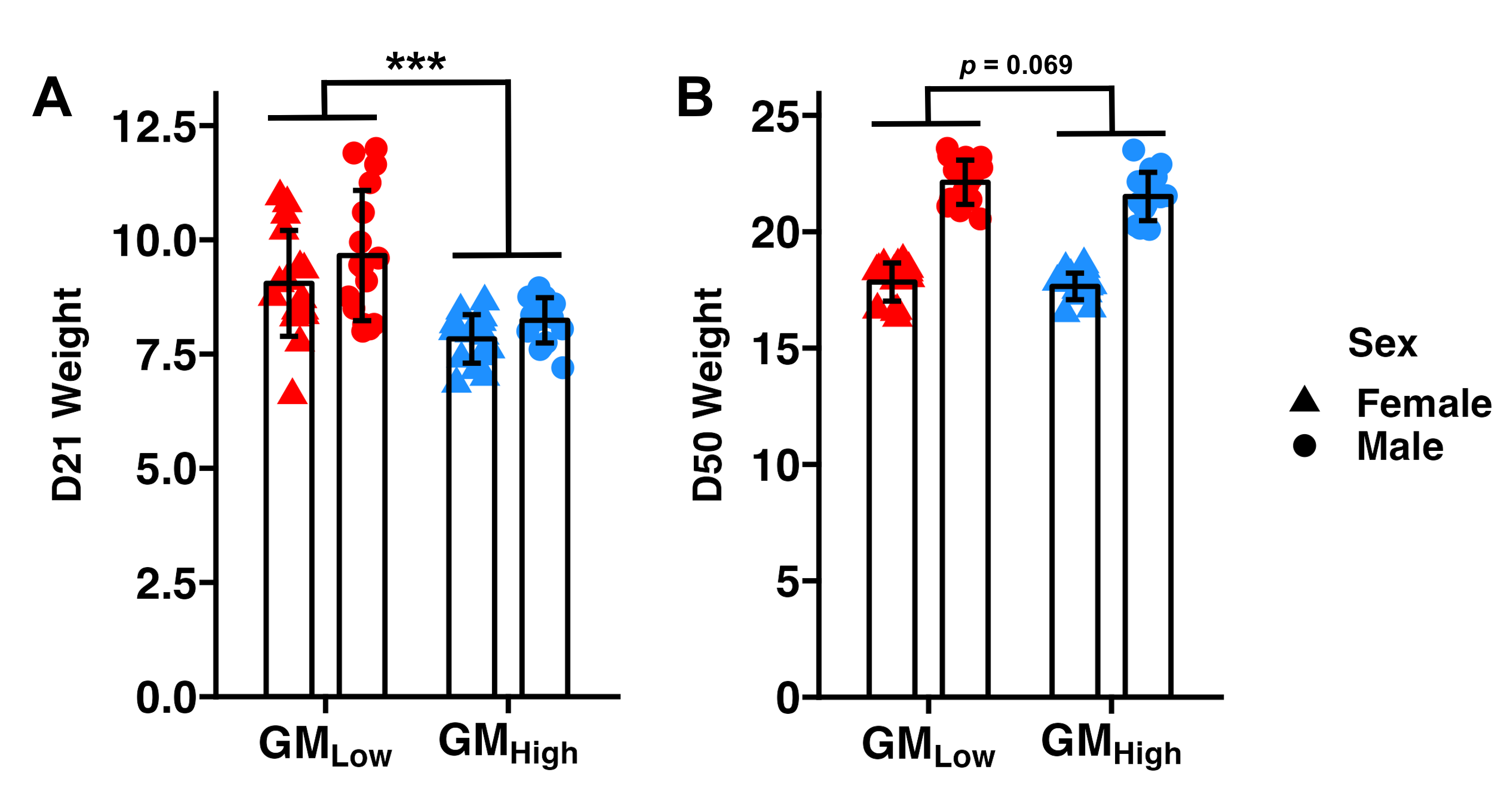

Supplement: Supplemental Material [file KGMI_A_2385524_SM3678.zip › Figure S3.tiff]

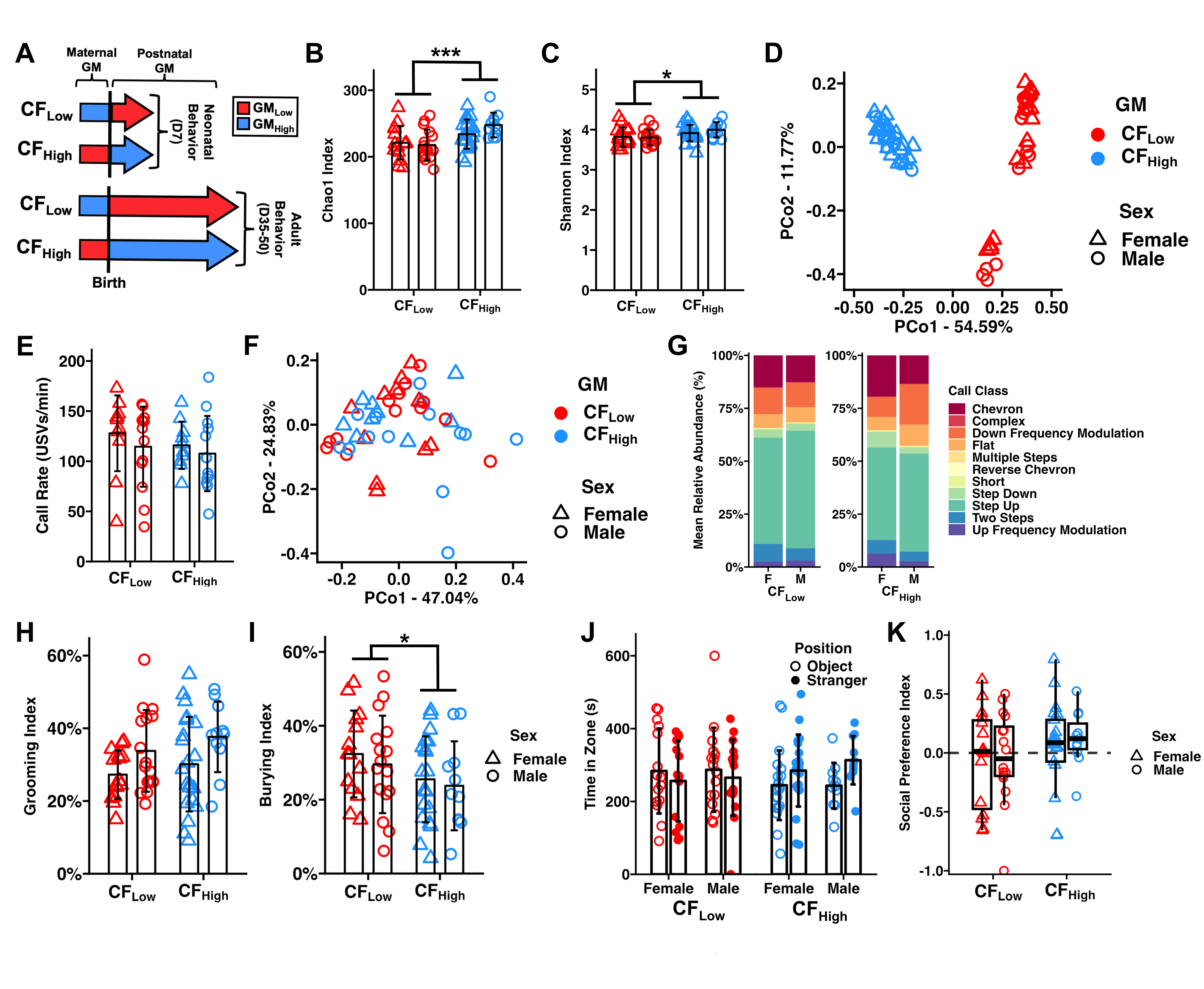

Supplement: Supplemental Material [file KGMI_A_2385524_SM3678.zip › Figure S4.tiff]

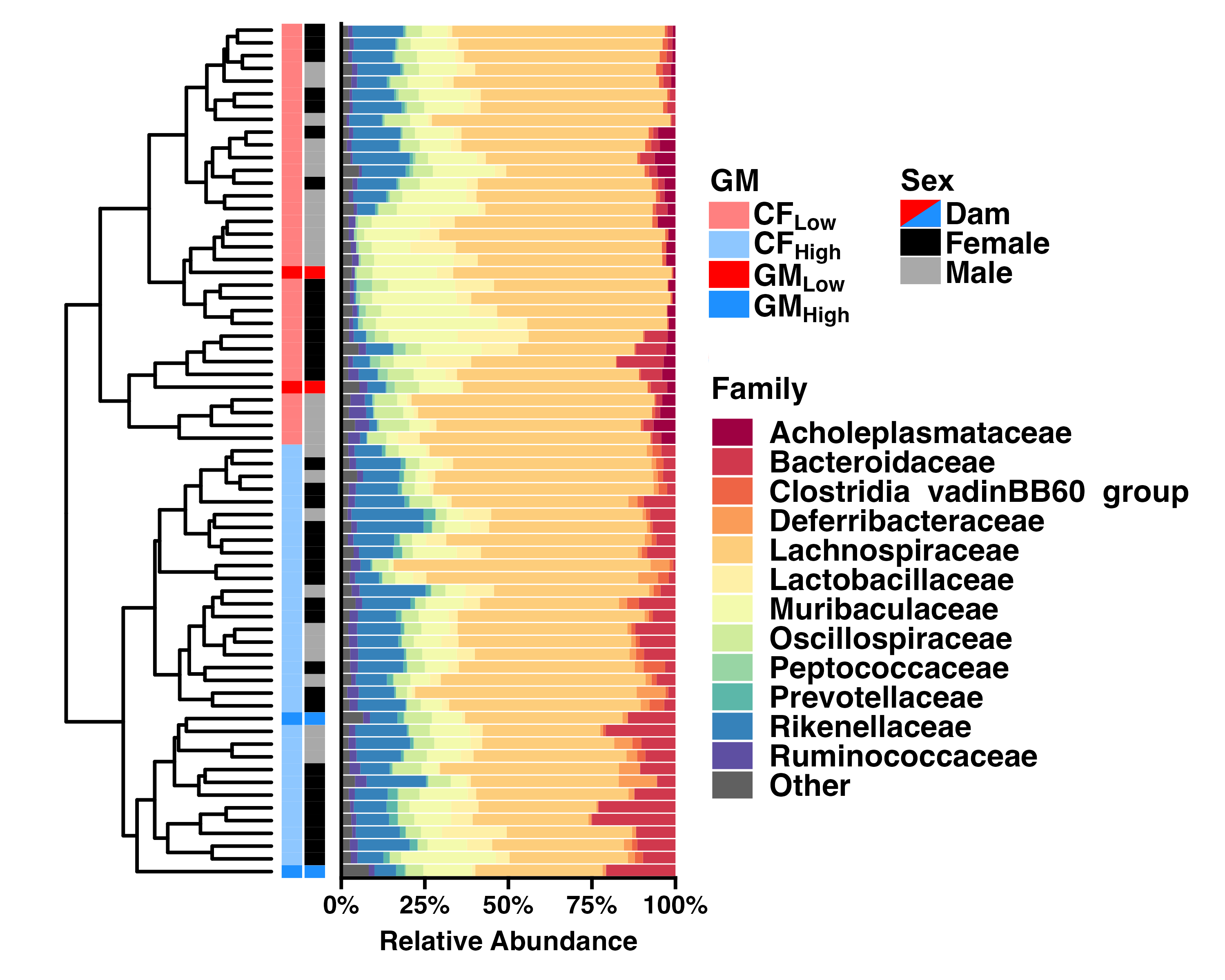

Supplement: Supplemental Material [file KGMI_A_2385524_SM3678.zip › Figure S5.tiff]

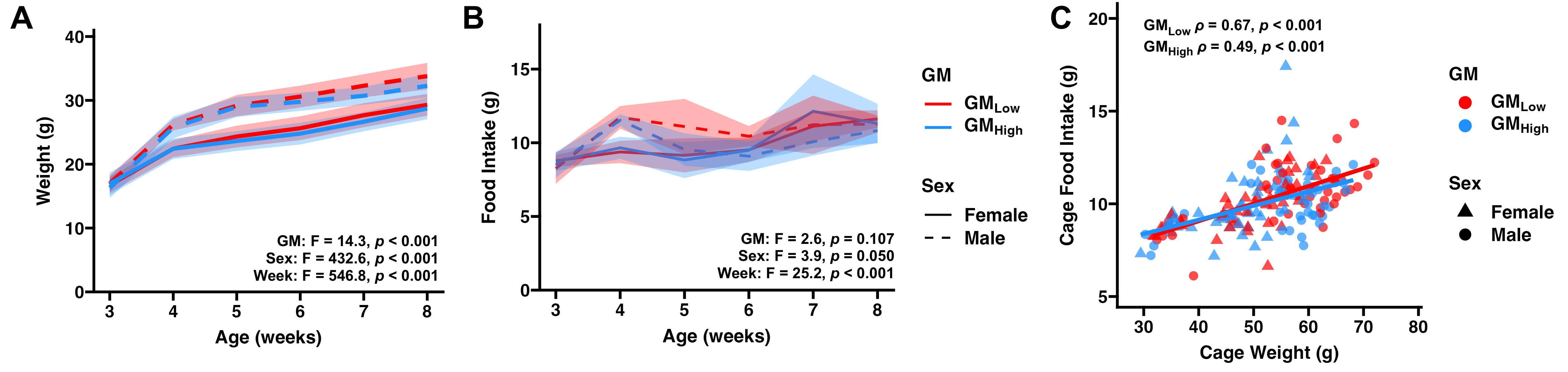

Supplement: Supplemental Material [file KGMI_A_2385524_SM3678.zip › Figure S6.tiff]
